# Inflammation in aortic surgery: postoperative evolution of biomarkers according pathologies and segments of the aorta

**DOI:** 10.1186/s13019-024-02672-4

**Published:** 2024-04-17

**Authors:** Martin T Yates, Alexander Smith, Alina A Mistirian, Carola M Bigogno, Michelle Lee, Ana Lopez-Marco

**Affiliations:** 1 Department of Cardiothoracic Surgery, St Bartholomew’s Hospital, West Smithfield, London, EC1A 7BE UK; 2 William Harvey Institute, Queen Mary University, London, UK

**Keywords:** Aortic surgery, Inflammation, Perioperative management

## Abstract

**Objectives:**

Aortic pathologies often present with elevated inflammatory biomarkers due to the nature of the disease. Open aortic surgery causes significant trauma to the body due to often mandatory ischemic periods, long cardiopulmonary bypass times and polytransfusion. We aim to determine postoperative trends on inflammation biomarkers for different aortic pathologies and type of surgery in different segments of the aorta.

**Methods:**

Retrospective review of prospectively collected data of 193 consecutive patients who underwent aortic surgery in our centre between 2017 and 2021, grouped according to the type of aortic intervention: (1) Type A aortic dissection (AD) repair with ascending aorta/hemiarch replacement, (2) Aortic root replacement (ARR), (3) Aortic arch + Frozen elephant trunk (FET), (4) Descending thoracic aorta (DTA)/Thoraco-Abdominal aortic repair (TAA). Primary outcomes were daily values of white blood cells (WBC) and C-Reactive Protein (CRP) during the first 15 postoperative days.

**Results:**

All groups had a similar inflammatory peak in the first 2–4 days (WBC 12-15 × 109 c/L). AD and FET groups show similar trends with WBC and CRP peaks on days 2 and 10. The ARR group didn’t experience the 2nd peak as most patients were already discharged. DTA/TAA patients experienced a more prolonged inflammatory response, reaching a plateau by day 5–10. AD group shows the highest WBC levels and the DTA/TAAA group the highest CRP levels. CRP levels remain elevated (100–200 mg/L) in all groups after 15 postoperative days.

**Conclusions:**

Inflammatory biomarkers show different postoperative trends depending on the clinical presentation and complexity of the aortic procedure performed. Further understanding of the inflammatory response to different aortic pathologies and surgical procedures will permit reduction on the liberal use of antibiotics that this cohort of patients are usually exposed to. An earlier version of the data included in this manuscript was presented as Oral Abstract in the UK Society of Cardiothoracic Surgery Annual meeting in 2021

## Introduction

Open aortic surgery poses a major insult to the body due to the associated long cardiopulmonary bypass and ischaemia to different organs; this translates into a general inflammatory response that it is usually exarcerbated by the need of polytransfusion in the perioperative period [1]. Furthermore, aortic pathologies often present with elevated inflammatory markers due to the nature of the disease (i.e. expanding aneurysm or acute aortic syndromes) [2]. 

Elevated inflammatory markers are often used as a surrogate for infection initiating antibiotic therapy, imaging and invasive investigations with further impacts in hospital stay.

There is little literature describing normal trends in inflammatory makers following major aortic surgery. We aim to determine normal postoperative trends on inflammatory markers for different aortic pathologies and anatomical segments of the aorta.

## Patients and methods

This study was approved by Barts Health NHS Trust Clinical Effectiveness Unit (ID:12,184) 26th May 2021.

Retrospective analysis of prospectively collected data for all consecutive patients undergoing aortic surgery in our institution from 2017 to 2021. Patient demographics, comorbidities, clinical presentation, operative data and postoperative complications were recorded in our local database (Dendrite Clinical Systems Ltd, UK) (Table 1).

**Table 1:** Demographics and outcomes of patients by operative group

	AD *n* = 50	ARR *n* = 45	FET *n* = 47	DTA/TAA *n* = 51	p value
Age	65.7 ± 12.2 (39–89)	49.2 ± 18.1 (20–87)	60.5 ± 14.4 (28–79)	53.1 ± 15.5 (18–78)	0.00001
Male sex	29 (58%)	33 (74%)	32 (68%)	31 (61%)	0.41
Elective	-	37 (83%)	26 (55%)	34 (67%)	
Urgent	5 (10%)	8 (18%)	15 (32%)	14 (27%)	
Emergency	45 (90%)	-	-	-	
CPB (mins)	203.5 ± 44.2	201.8 ± 55.9	325.7 ± 72.8	N/A	0.0001
XC (mins)	101.0 ± 34.3	153.1 ± 44.9	189.9 ± 65.5	N/A	0.0001
CA (mins)	36.2 ± 15.7	-	89.1 ± 52.5	N/A	0.0001
Temperature	21 (42%)	26 (58%)	24 (50%)	33 (65%)	0.12
Positive culture	18 (36%)	2 (4%)	9 (19%)	8 (16%)	0.001
Antibiotics	38 (76%)	23 (51%)	40 (83%)	44 (86%)	0.0002
Days Antibiotics	6 ± 4.6 (0–15)	5.4 ± 7.8 (0–42)	9 ± 8 (0–39)	7.5 ± 4.4 (0–15)	0.03
Tracheostomy	8 (16%)	1 (2%)	12 (25%)	4 (8%)	0.006
Haemofiltration	13 (26%)	2 (4%)	12 (25%)	23 (45%)	0.0001
In-hospital mortality	7 (14%)	3 (7%%)	5 (11%)	3 (6%)	0.48
Length of stay (days)	18.9 ± 9.1 (5–43)	13 ± 17 (5-110)	27 ± 19 (6–78)	20 ± 11 (8–48)	0.0001

AD: Aortic dissection, ARR: Aortic root replacement, FET: Frozen elephant trunk, DTA/TAA: Descending thoracic aorta / thoracoabdominal aorta, CPB: cardiopulmonry bypass time, XC: cross clamp time, CA: circulatory arrest

Patients were grouped according to the type of aortic intervention in 4 categories: (1) Type A aortic dissection (AD) with repair limited to ascending aorta/hemiarch, (2) Aortic root replacement (ARR) for elective aneurysms, (3) Aortic arch + Frozen elephant trunk (FET) for elective aneurysms and/or chronic dissections and (4) Descending thoracic aorta (DTA)/Thoraco-Abdominal aortic repair (TAA) for elective aneurysms and/or chronic dissections.

Primary outcomes were daily values of white blood cells (WBC) and C-Reactive Protein (CRP) during the first 15 postoperative days. Secondary outcomes were use of antibiotics, fever (measured as > 38 °C) and positive blood, urine or sputum cultures.

### Statistical analysis

Continuous variables are expressed as mean ± standard deviation (SD) as the data showed normal distribution. Comparison between groups was performed with ANOVA test. Categorical variables are expressed as percentages and compared using Chi square test.

A longitudinal analysis adjusted by time and individual groups was performed. Multivariable regression modelling was undertaken to assess the association between post-operative inflammatory markers and the aortic procedure undertaken, adjusted for patient sex, time, and baseline marker level.

A saturated mixed-effects model was fitted with the outcome. Sex, time and centred baseline markers were fitted into the model, as well as all of their interaction terms and a covariance structure corresponding to a random intercept for patients and slope for time. The model where the level 1 errors varied with procedure category fitted the data better than the model where they were assumed to have the same dispersion (likelihood-ratio test *p* < 0.0001). The fixed effect structure was then simplified using the Wald test, maintaining hierarchy of terms by assessing the significance of polynomial terms and the most complex interaction terms first. Following this sex and baseline markers were excluded from the model. The model assumptions were that (a) missing data are at random, (b) the model is correctly specified, (c) within-cluster variation was independent of between-cluster variation, (d) between-cluster variation was independent of the predictor variables, (e) random intercepts and slope were independent of one another, (f) between cluster-residuals are conditional on cluster-level and elementary level variables, and are ∼ NID(0, σ_u_^2^), and (g) within-cluster residuals are conditional on cluster-level residuals, cluster-level predictor variables, elementary-level predictor variables, and are ∼ NID(0, σ_e_^2^).

The data was analysed using STATA 22.

### Surgical technique

Patients in groups 1 to 3 are approached via median sternotomy and surgery is conducted with cardiopulmonary bypass and cardioplegic cardiac arrest. Systemic circulatory arrest and cerebral protection are mandated for groups 1 and 3, but only those on group 2 requiring hemiarch replacement. Bladder temperature is maintained at 22–24 °C for groups 1 and 3 and 28–32 °C fro group 2 depending on the need to perform an open distal anastomosis (hemiarch).

Patients in group 4 are approached by thoracolaparotomy and surgery is conducted with left heart bypass, sequential clamping and intermittent spinal and visceral ischemia. Bladder temperature is maintained at 34 °C.

Our institutional protocol includes intraoperative administration of weight adjusted doses of platelets, fibrinogen and fresh frozen plasma or prothrombin concentrate as well as further doses and other products such as red blood cells and/or cryoprecipitate based on thromboelastography and/or formal blood count results.

### Radiological imaging

Our institutional protocol includes transthoracic echocardiogram and contrast-enhance CT aortogram to be performed for all this patients prior to discharge. Serial chest-X rays are done after insertion of central lines, removal of chest drains and guided by clinical symptoms.

## Results

A total of 193 patients were included in the study and grouped as follows: (1) AD *n* = 50, 25.9%, (2) ARR *n* = 45, 23.3%, (3) Arch/FET *n* = 48, 24.4% and (4) DTA *n* = 51, 26.4%.

Mean age was 57.3 ± 15.1 years (18–89) and the majority of patients were male (*n* = 125, 65%). Patients in the ARR and DTA/TAA were significantly younger than in the other two groups, accounting for connective tissue disorders.

Patients in group 1 were operated mostly as emergency due to nature of their disease, whilst the other 3 groups combined different timings for surgery, but mostly elective indications.

In terms of operative strategies, groups 1–3 were operated with cardiopulmonary bypass (CPB) and aortic cross clamp (XC) with circulatory arrest (CA) when needed to conduct an open distal anastomosis near or within the arch. AD repair with ascending and hemiarch replacement had the shorter CPB and CA times, while arch/FET associated longer CPB and CA times (*p* < 0.05).

Patients in group 4 were operated using left heart bypass with sequential cross clamping and ischemia to individual teritorries.

### Individual trajectories for WBC

Overall mean baseline WBC count was 8.5 ± 2.8 × 10^9^/L and by groups: (1) AD, 11.1 ± 3.4 × 10^9^/L, (2) ARR, 6.9 ± 1.4 × 10^9^/L, (3) FET 7.9 ± 2.2 × 10^9^/L, (4) DTA/TAAA, 7.7 ± 2.5 × 10^9^/L.

**Fig. 1:**
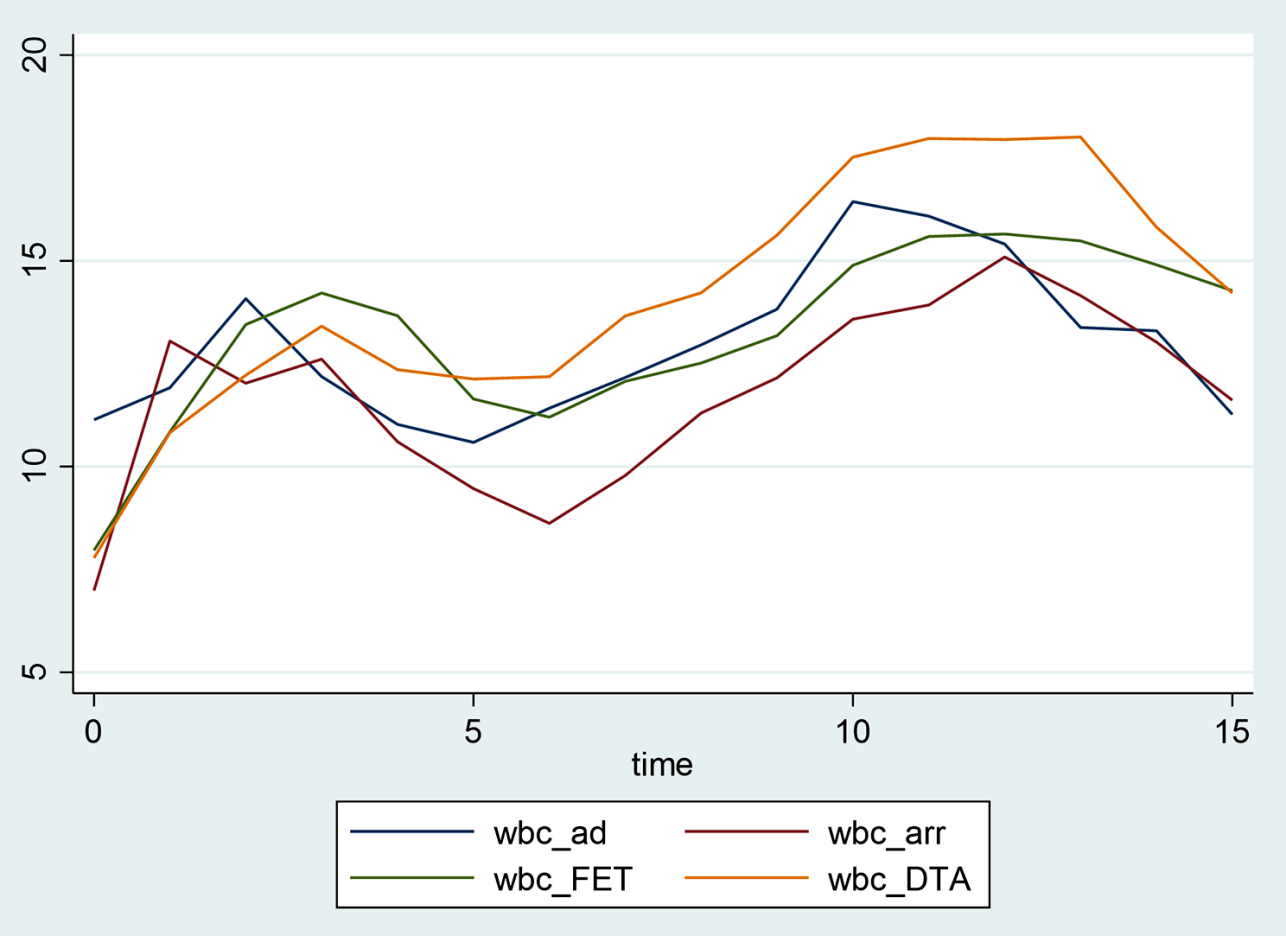
Mean trend of white blood cell count (unit x 10^9^/L) during postoperative stay (time measured in days) in different groups according segment of the aorta operated on

**Fig. 2:**
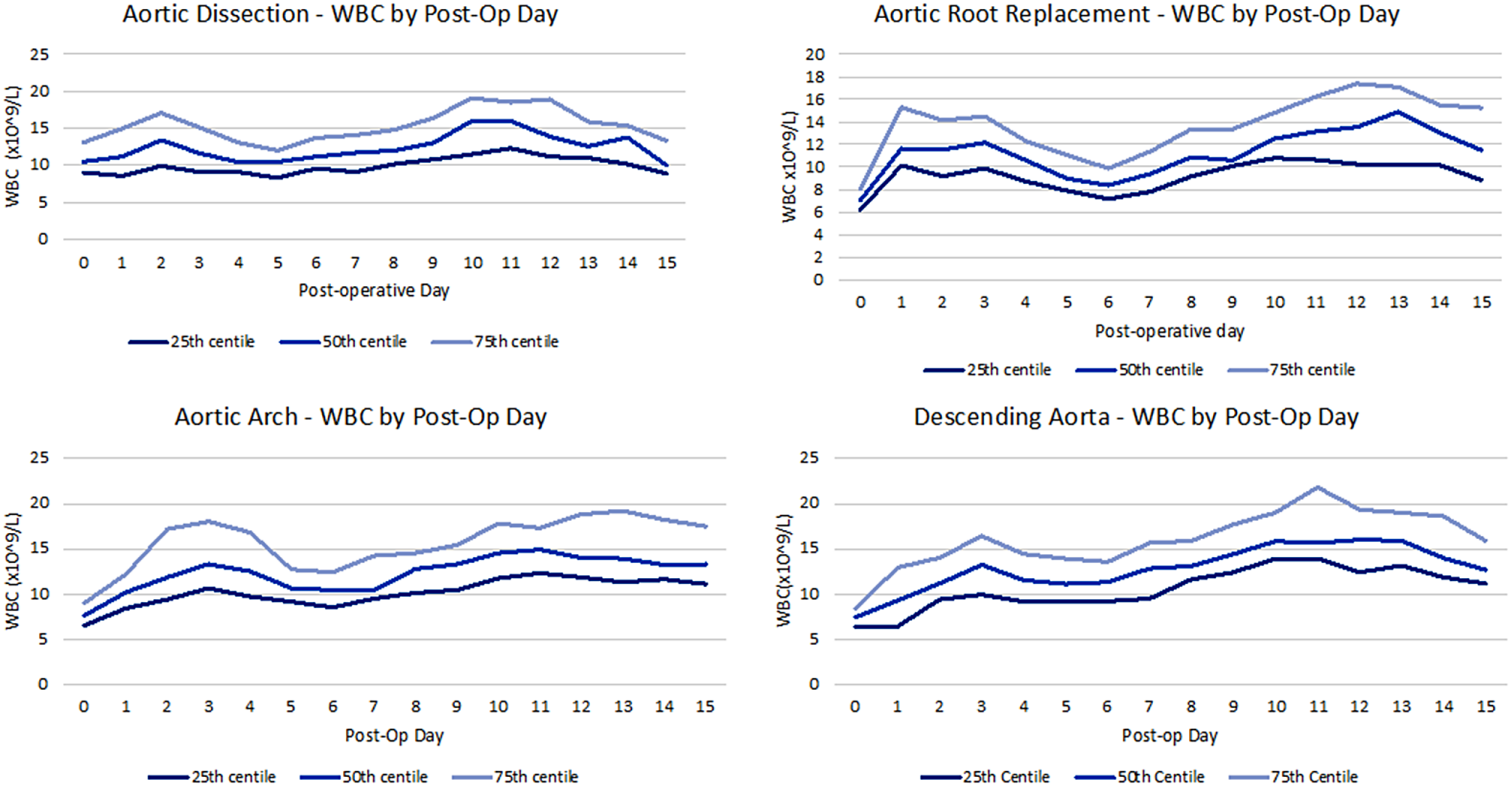
Trend of white blood cell count (expressed as median and interquartile range in unit x 10^9^/L) for each postoperative day in different groups according segment of the aorta operated on

Following surgery the mean WBC trajectories evidence a similar rising tendency for all groups with two similar peaks around day 2–3 and day 10 (Fig. 1). However, looking at the graphs for individual WBC trajectories within each group, we observe that patients with AD have the higher baseline mean WBC count (11.1 ± 3.4 × 10^9^/L) and experienced a inflammatory peak on day 2 (mean WBC 14.1 ± 5.5 × 10^9^/L) and then on day 10 (mean WBC 16.4 ± 9.0 × 10^9^/L). Patients undergoing elective aneurysm repair experienced inflammatory peaks at different days depending on the segment of the aorta operated on: day 1 (13.0 ± 4.6 × 10^9^/L) and 12 ( 15.1 ± 6.2 × 10^9^/L) for ARR; day 3 (14.2 ± 5.2 × 10^9^/L) and 12 (15.6 ± 4.8 × 10^9^/L) for FET and day 3 (13.4 ± 5.1 × 10^9^/L) and 13 (18.0 ± 8.5 × 10^9^/L) for DTA/TAA (Fig. 2).

The mixed effects regression model showed extremely strong evidence that after adjusting for time and baseline, FET and DTA/TAA surgeries were associated with significant WBC rise compared to the other procedures (2.3 × 10^9^/L rise in FET and 3.1 x x10^9^/L rise in DTA/TAA, *p* < 0.001). There was no difference accountable to sex, age or positive cultures (blood, urine or sputum).

### Individual trajectories for CRP

Overall mean baseline CRP was 21.5 ± 16.5 mg/L and by groups: (1) AD, 22.3 ± 13.7 mg/L, (2) ARR, 22.3 ± 13.7 mg/L, (3) FET 25.1 ± 17.2 mg/L, (4) DTA/TAAA, 23.8 ± 17.4 mg/L.

**Fig. 3:**
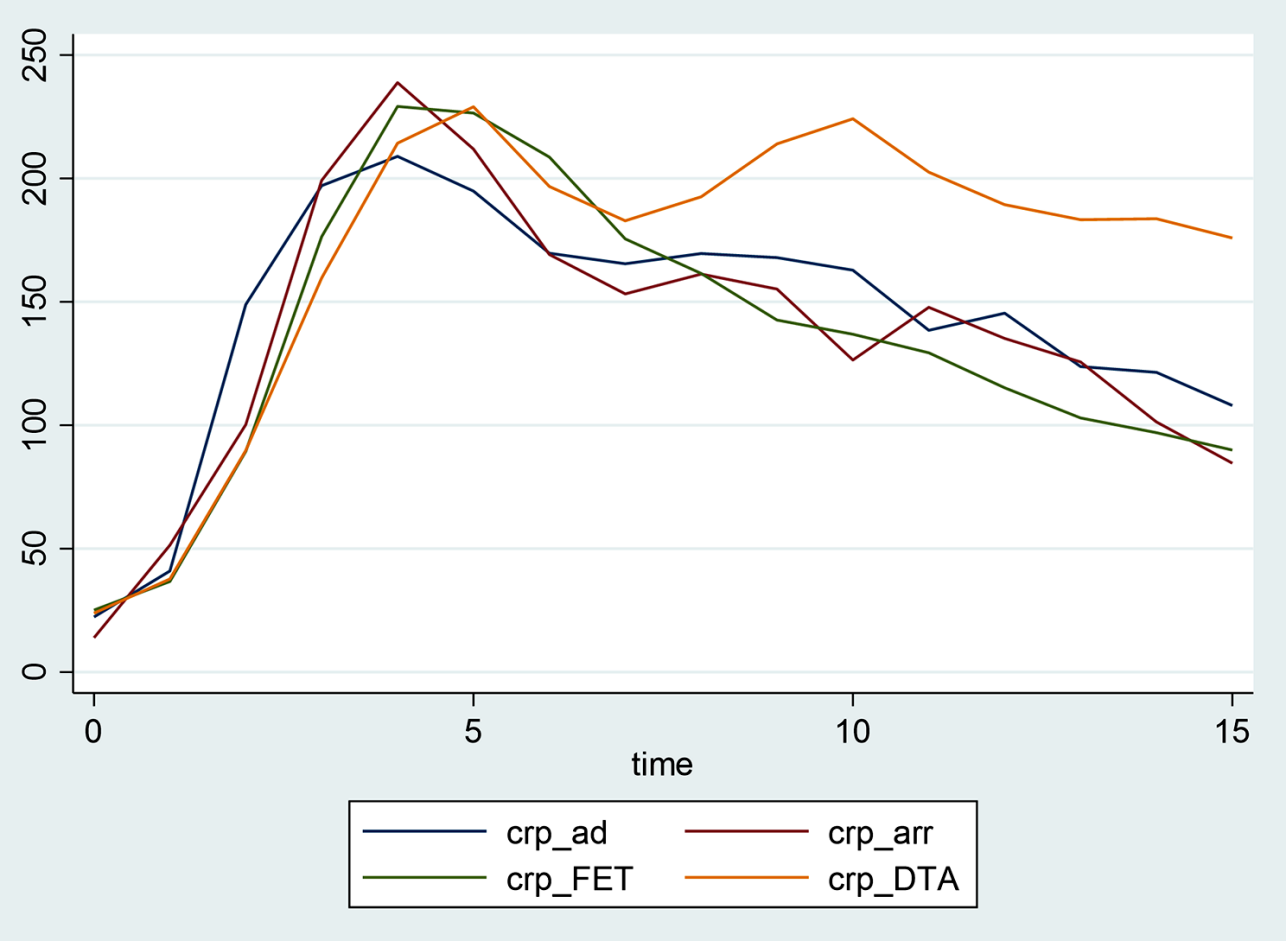
Mean trend of C-Reactive Protein levels (µg/mL) during postoperative stay (time measured in days) in different groups according segment of the aorta operated on

**Fig. 4:**
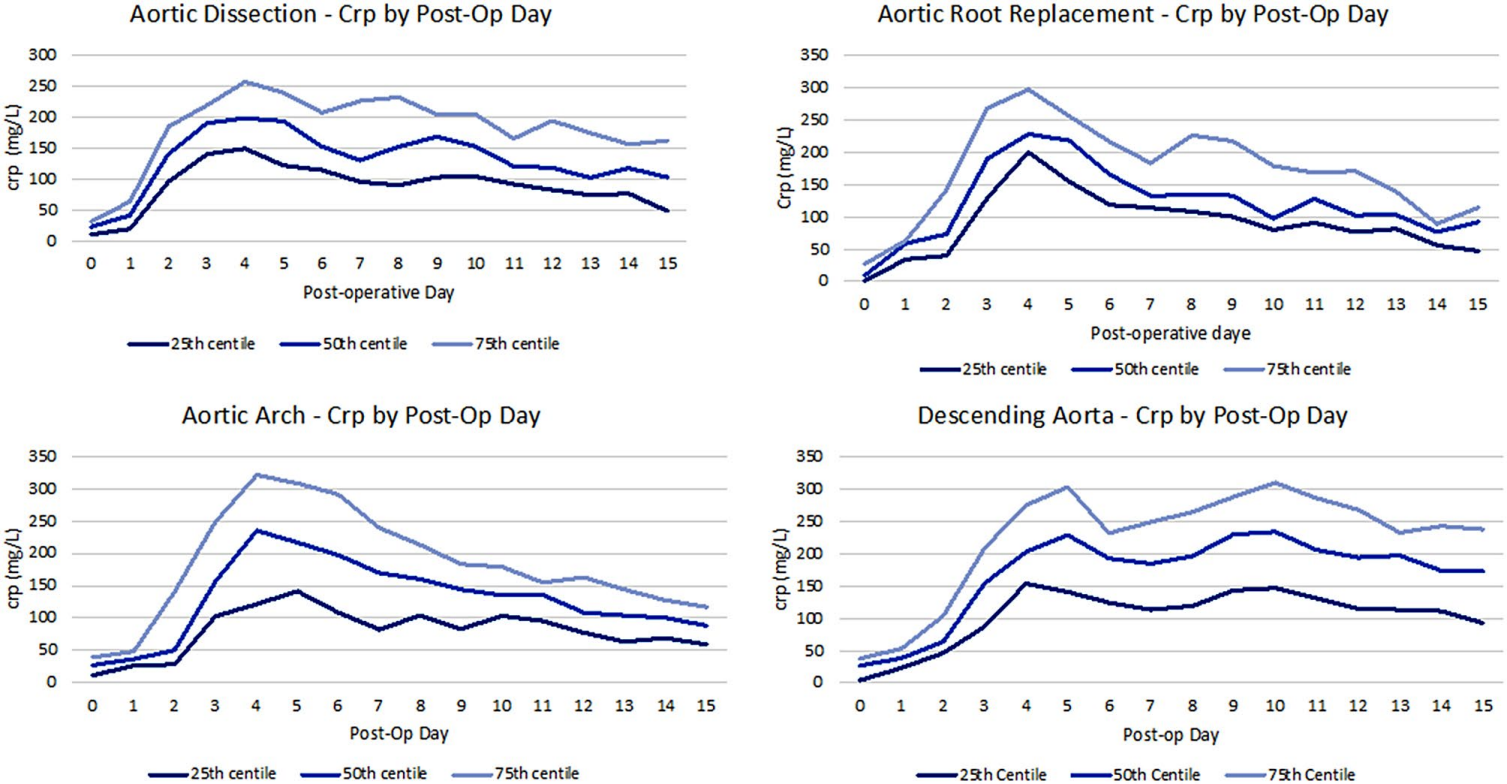
Trend of C-Reactive Protein levels (expressed as median and interquartile rand in µg/mL) for each postoperative day in different groups according segment of the aorta operated on

Following surgery the mean CRP trajectories evidence a similar rising tendency for all groups with a similar peaks around day 4 (Fig. 3). After that, the CRP decreases progressively in the following day towards nomal values, similarly for all groups although the DTA/TAA group shows a longer plateau of elevated CRP for the first 2 postoperative weeks. Looking at the graphs for individual CRP trajectories within each group, we observe that patients undergoing arch/FET have the higher baseline mean CRP value (25.1 ± 17.2 mg/L). the highest CRP values are seen in postoperative day 4 for all groups (248.1 ± 288.5 mg/L for AD and ARR groups, and 229.1± 120 mg/L for arch/FET and DTA/TAA). Patients undergoing DTA/TAA repair have a prolonged elevated CRP throughout the first 15 postoperative days, with a mean CRP value of 175.9 ± 84.6 mg/L by day 15 (Fig. 4).

The mixed effects regression model showed extremely strong evidence that after adjusting for time and baseline, DTA/TAA surgery was associated with significant CRP rise compared to the other procedures (23.3 mg/L rise, *p* = 0.02). There was no difference accountable to sex, age or positive cultures (blood, urine or sputum).

### Use of antibiotics and clinical correlation

Overall, 145 patients (75.1%) were given antibiotics in the postoperative period. There was variation amongst the groups, with patients in the AD, arch/FET and DTA/TAA groups receiving significantly more treatment with antibiotics thant the ARR group, *p* < 0.05 (1) AD 38, 76%; 2) ARR 23, 51%; 3) FET 40, 84% and 4) DTA/TAA 44, 86%).

During their hospital stay, 104 patients (72%) had a temperature spike over 38 C with no difference amongst the groups: (1) AD 21 (42%), (2) ARR 26 (58%), (3) FET 24 (50%) and (4) DTA/TAA 33 (65%). Only a minority of patients (37; 26%) had a confirmed positive culture of blood, sputum or wound, predominantly in groups 1) AD 18 (36%), 3) FET 9 (19%), and 4) DTA/TAA 8 (16%) *p* = 0.001. The length of antibiotic administration was also significantly longer in groups 3 (9 ± 8 days) and 4 (7.5 ± 4.4 days), *p* = 0.03.

Length of stay was significantly longer for groups 3 and 4 (27 ± 19 and 20 ± 11 days respectively), intermediate for group 1 (18 ± 9.1 days) and shorter for group 2 (13 ± 17 days), with only 50% of the patients of the cohort being still admitted in hospital after day 7 in group 2 and day 10 in group 1 (*p* < 0.05) (Figs. 3 and 4).

## Discussion

This study describes the trends of inflammatory markers following aortic surgery. There was consistent rise in WCC and CRP with a peak between Day 2–4 seen in all groups. The peak tended to be higher in the dissection patients however their baseline was also higher initially.

Elective patients would be expected to have normal inflammatory markers pre operatively due to the stringent assessment carried out to prevent operating of patients with occult infections. In the aortic dissection group however they often had raised inflammatory markers at presentation, even with the bloods taken within hours of the initial pain. This can be explained by the pathophysiology of acute aortic syndroms with an intimal tear propagating rapidly along the length of the aorta followed by the development of haemoatoma between the layers of the aortic wall [3]. In some cases extravasation of blood into the pericardium may also occur. This combination of events likely initiates and maintains the systemic inflammatory response. This study has shown that such a response can be measured on arrival at hospital, within hours of the acute event.

Differential inflammatory responses can be seen among the 4 groups. These can most likely be explained by variations in the nature of aortic pathology (i.e. acute aortic dissection vs. stable aneurysms or chronic dissection) and the extent of surgery required for the different segments of the aorta, which determines the systemic temperature used and the quite varied duration of CPB and CA times. It is well recognised that the CPB duration is directly responsible of the systemic inflammatory reaction that occurs within the body.

It is also known that intestinal ischemia-reperfusion occurring during CPB induces a systemic inflammatory reaction. Prolonged CPB could increase intestinal permeability and this lead to bacterial tranlocation from the instestine to the bloodstram. This effect is potentially more common in patients undergoing TAAA repair, where the visceral flow is not only discontinued by altered by artificial perfusion from the bypass machine.

Amongst out groups, aortic root replacement had shorter CPB and XC times with no need for circulatory arrest in the majority of cases. These patients usually recover faster with less than half of the patients in our cohort still in hospital after day 7.

The highest inflammatory response was associated with Arch/FET and DTA/TAA groups. Significantly higher WBC count was seen in these groups with prolonged, maintained CRP rise following DTA/TAA. The extent of these operations explains much of this inflammatory response. Arch/FET mandates prolonged CPB and XC times with circulatory arrest compared to the other groups; DTA/TAA, on the other hand, requires an extensive thoracolaparotomy incision with intermittent ischemia to multiple vital organs. The consequences of this is often a coagulopathy mandating administration of multiple blood products for the first postoperative days to correct coagulopathy and maintain Hb above 9 g/L; transfusion in itself know to induce inflammation [4]. 

Post operative infection following cardiac surgery is associated with significant morbidity and mortality and also healthcare costs. Sternal wound infection prolongs hospital stay, requires intravenous antibiotics and multiple wound debridements in theatre, occasionally culminating in reconstructive surgery [5]. Aortic surgery often involves implantation of prosthetic valves and grafts therefore bacteramia can have devastating consequences if these are seeded with infection [6]. However, antibiotic therapy must be appropriate and targeted to prevent resistance and side effects from therapy.

Despite the low rate of confirmed infection by positive cultures among the groups, a high percentage of patients were administered antibiotics to treat either isolated temperature spikes or most frequently, empirically to treat the raised markers. The length of treatment with antibiotics was often a week or more which likely contributed to a significant increase in hospital stay.

With the majority of aortic patients experiencing significant and sustained rises in inflammatory markers that we have shown in this study, priority must be placed on clinical assessment and imaging to distinguish between inflammation and infection. Trials with anti-inflammatory drugs such as Colchicine or Ibuprofen have been used in selected cases, with no demonstrated infection and after balancing risks or postoperative bleeding. We would not recommend routinely the use of anti-inflammatory drugs as they associate their own risks (i.e. acute renal failure, increasing risk of bleeding). The use of steroids is also not recommended routinely as it will increase significantly the risk of perioperative infection, not to forget the negative impact in healing; in nay case, the administration of steroids is associated with increasing WBC values on its own.

We hope to reduce the liberal administration of antibiotics in these patients by describing the natural inflammatory response to aortic surgery.

Limitations of our study include the concious selection bias of only selecting AD patients who required aortic repair only limited to the ascending aorta in an attempt to avoid mixing pathologies and segements of the aorta. We aknowledege that limiting the inflammation analysis to WBC and CRP values is quite simplistic, yet those are parameters that are widely available in the day to day practice.

## Conclusion

Inflammatory biomarkers show different postoperative trends depending on the clinical presentation and complexity of the aortic procedure performed. Further understanding of the inflammatory response to different aortic pathologies and surgical procedures will permit reduction on the liberal use of antibiotics that this cohort of patients are usually exposed to.

## Data Availability

Data will be available upon request.
